# Ifosfamide ±VP16 in Childhood' Malignancy

**Published:** 1988

**Authors:** B. Pizer, A. Oakhill, M. G. Mott, G. Sleight

**Affiliations:** Bristol Children's Hospital; Bristol Children's Hospital; Bristol Children's Hospital; Bristol Children's Hospital


### Ifosfamide ±VP16 in Childhood' Malignancy: A Phase II (toxicity) Study

**B. Pizer, A. Oakhill, M. G. Mott and G. Sleight**
Bristol Children's Hospital

This paper reports the toxicity associated with Ifosfamide therapy in the treatment of childhood malignancy. 30 children with solid tumours received 92 treatment courses. Ifosfamide (6 gms/m$^2$) was given as a single agent to 16 patients (46 courses) and in combination with VP16 (213), 150 mg/m$^2$ daily for 3 days, to 14 patients (46 courses). MESNA was given concurrently in one of two regimens to prevent haemorrhagic cystitis.

Myelosuppression was universal but recovered by day 22 in 81 out of 88 evaluable courses. It was greater in the combined therapy group. Mean haemoglobin fall was 1.35 gm/dl for the single agent group and 1.91 gm/dl in the combined therapy group. 10 transfusions were given, all to patients receiving additional VP16. Nadir neutrophil counts fell below $0.5 \times 10^9$/L in 42% of the single agent group and 69% of the two agent group. There were no documented bacteraemias. Nadir platelet count fell below $50 \times 10^9$/L in 6 cases (combined therapy); only one platelet transfusion was required, for a minor bleeding episode.

There were no episodes of frank haematuria. The higher dose of MESNA did not confer protection against microscopic haematuria. Emesis occurred in all but two patient courses, but was considered of comparable severity to other non-platinum containing regimens. Two patients had a generalized convulsion, both with full recovery. There was no demonstrable renal or hepatic toxicity.

We conclude that these regimens have acceptable toxicity in the context of the high risk patients treated and the alternative available treatment options.

### INTRODUCTION

Alkylating agents are of major importance in the treatment of malignant solid tumours in childhood. The efficacy and toxicity of one such agent, cyclophosphamide (an oxazophorine) have been widely reported. Ifosfamide, another drug of the oxazophorine class, has been shown to be active against a wide range of human malignancies in both adults and children. There is evidence to suggest that Ifosfamide and Cyclophosphamide have significant differences in their modes of action, spectrum of activity and toxicity (1). Phase II trials using ifosfamide began in the early 1970's but its use was restricted until recently by the major dose-limiting side-effect of urothelial toxicity. Recent studies have shown that the thiol compound Sodium 2 mercaptoethane sulphonate (MESNA) protects the urothelium from the toxic effects associated with high dose oxazophorine therapy, probably by combining with and inactivating their toxic metabolites, particularly acrolein.

Since November 1983, Ifosfamide has been incorporated in some of our first-line chemotherapeutic regimens in the treatment of solid malignancies of childhood. This paper reports the toxicity experienced in the first thirty children treated by us with Ifosfamide (92 courses) when used both as a single agent and in combination with the epipodophyllotoxin VP16(213) (Table 1). The response rates to these regimens will be reported elsewhere.

**Table 1**

**November 1983–December 1985**

| | Number Patients | Number Courses |
|---|---|---|
| Ifosfamide | 16 | 46 |
| Ifosfamide+VP16 | 14 | 46 |
| TOTAL | 30 | 92 |

### Patients and Methods

A broad range of solid tumours was treated (Table 2). There were 19 boys and 11 girls with an age range of 2–18 years, (median 8.5). None had had previous pelvic radiation. Sixteen received a total of 46 courses of Ifosfamide as a single agent, while 14 received combination therapy for a total of 46 courses (Table 1). Most courses were given as part of established chemotherapy protocols involving 3 week treatment cycles in rotation with other established agents.

**Table 2**

**Diagnosis**

| | |
|---|---|
| Neuroblastoma | 7 |
| Ewings sarcoma | 5 |
| Osteosarcoma | 4 |
| Rhabdomyosarcoma | 4 |
| SRCT –Small Round Cell Tumour | 3 |
| Others | 7 |

A full clinical history and examination were undertaken before and after each course. Full blood count, liver function tests and plasma electrolytes, urea and creatinine were estimated with each course. Pre-treatment intravenous hydration was given routinely as 2.5% dextrose/0.45% saline (100 ml/m$^2$/hour) for at least 3 hours. VP16 was given at a standard dose of 150 mg/M$^2$/day for three days, the first dose being given prior to the administration of ifosfamide, diluted in 100 mls of 0.9% saline and infused over 60 minutes. Ifosfamide was given at a standard dose of 6 G/M$^2$ diluted with MESNA in 500 mls of dextrose/saline and infused over 24 hours. A second infusion of dextrose/saline was given concurrently to provide overall hydration at a rate of 100 mls/m$^2$/hour. Post-hydration was given as dextrose/saline at 100 mls/m$^2$/hour for a variable period (range 14–34 hours), depending on the degree of nausea and vomiting with each course.

Various MESNA regimens were tried but only courses using the two latest regimens of MESNA administration are reported here (Table 3). The dose for the continuous 24-hour infusion was given in the same bag as the Ifosfomide. The final bolus dose of MESNA was given orally if tolerated. All urine passed during admission was collected, inspected for gross haematuria and tested with Labstix (AMES) to detect microscopic haematuria. All positive tests for blood or haemoglobin were recorded as positive (Range trace to 3+) microscopic haematuria and scored as shown in table 8. Antiemetic therapy was given routinely to all patients unless refused, the usual regimen being alternating iv Chlorpromazine and Promethazine at doses of 0.5 mg/Kg. The frequency and volume of emesis was recorded throughout hospitalisation.

**Table 3**

**Treatment regimens**

*Regimen A:* Ifosfamide with MESNA, 6 G/m$^2$/24 hr+ MESNA, 1 G/m$^2$ at −1, +27, +30, +33 hr, +36 hr

*Regimen B:* Ifosfamide, 6 G/m$^2$/24 hr with MESNA, 9 g/m$^2$ /24 hr MESNA 1.5 G/m$^2$ at −1, +27, +30, +33 & +36 hr.

*Regimen C:* Same as Regimen A +VP16 150 mg/m$^2$/d×3 days

*Regimen D:* Same as Regimen B +VP16 150 mg/m$^2$/d×3 days

All patients were seen routinely at or around the 10th day post chemotherapy for full history and examination and nadir FBC. A further FBC was performed prior to the next course of chemotherapy to ensure recovery from myelosuppression—i.e. neutrophils>1×10$^9$/1 and platelet count>100×10$^9$/1 and delays in administration of the next course of chemotherapy due to myelosuppression were recorded.

### RESULTS

### Toxicity

1. *Myelosuppression:*

   a) *Anaemia*—Four courses were unevaluable due to lack of data and 4 because of transfusions given at the time of chemotherapy (Table 4). Packed red cells were generally administered if the nadir haemoglobin concentration fell below 8G/dl. No patient receiving regimens A or B required transfusions, but they were required following 9 of 30 evaluable courses of regimen C and 1 of 11 evaluable courses of regimen D.

   b) *Neutropaenia*—The nadir neutrophil counts (×10$^9$/1) are shown in Table 5. A significant pyrexia (temperatures>38.5°C) associated with neutropaenia (<1×10$^9$/1) occurred after only 4 courses; 3 of regimen C and 1 of regimen A. All 4 remained culture negative and became afebrile on our standard iv antibiotic regimen (Cefuroxime and Tobramycin) with 4 days.

   c) *Thrombocytopaenia*—Significant nadir platelet counts (<100×10$^9$/1) are shown in Table 6. There was only one significant bleeding episode (epistaxis associated with thrombocytopaenia) on regimen C, which rapidly resolved following platelet transfusion.

   d) *Delay*—Myelosuppression resulted in delay of the next course of chemotherapy in only 7 of 88 evaluable courses (Table 7) 5 following regimen C (26–33 days and one each following regimens B and D.

2. *Haematuria* (Table 8)
   4 courses were unevaluable due to insufficient data. No frank or gross haematuria was observed throughout the study. Microscopic haematuria was found as detailed in the table.

3. *Other Toxicity*

   a) *neurological:* Two patients experienced generalized tonic-clonic convulsions whilst receiving chemotherapy, but recovered fully with no apparent sequelae. Chemotherapy was repeated in both patients using the same drugs/dosages without further seizure activity. Plasma electrolytes, osmolality,

**Table 4**

**Haemoglobin**

| | *Regimens A+B* | *Regimens C+D* |
|---|---|---|
| *No. of courses available* | 43 | 41 |
| Change in Haemoglobin (gm/dl) | | |
| Range | +1.1 to −3.0 | −0.4 to −3.5 |
| Median | −1.25 | −1.9 |
| Mean | −1.35 | −1.91 |

**Table 5**

**Neutropaenia**

| | *Reg. A* | *Reg. B* | *Reg. C* | *Reg. D* | *Total* |
|---|---|---|---|---|---|
| Courses | 21 | 25 | 34 | 12 | 92 |
| No. evaluable | 18 | 25 | 33 | 12 | 88 |
| Nadir anc ×10$^9$/L | | | | | |
| >1.5 | 1 | 4 | — | — | 5 |
| 1−1.5 | 1 | 2 | — | 1 | 4 |
| 0.5−1 | 10 | 7 | 8 | 5 | 30 |
| <0.5 | 6 | 12 | 25 | 6 | 49 |

**Table 6**

**Thrombocytopaenia**

| | *Reg. A* | *Reg. B* | *Reg. C* | *Reg. D* | *Total* |
|---|---|---|---|---|---|
| Courses | 21 | 25 | 34 | 12 | 92 |
| No. evaluable | 18 | 25 | 33 | 12 | 88 |
| Nadir | | | | | |
| 50–99,000 | 1 | 3 | 7 | 2 | 13 |
| <50,000 | 0 | 0 | 6 | 0 | 6 |
| Range | 65−467K | 55−341K | 19−622K | 60−267K | |
| Platelet Transfusion requirement | — | — | 1 | — | 1 |

**Table 7**

**Myelosuppression**

| | Nadir Counts (Day) Range | Mean | Recovery Counts (Day) Range | Mean |
|---|---|---|---|---|
| Reg. A | 7–19 | 11.1 | 16–26 | 20.2 |
| Reg. B | 7–15 | 10.5 | 16–25 | 21.2 |
| Reg. C | 8–14 | 10.4 | 15–33 | 21.8 |
| Reg. D | 9–12 | 10.1 | 15.29 | 21.0 |

**Table 8**

**Microscopic haematuria**

| | Reg. A | Reg. B | Reg. C | Reg. D |
|---|---|---|---|---|
| Incidence | 4/19<br>19% | 14/23<br>60.1% | 13/34<br>38.2% | 9/12<br>75% |
| Mean severity score: | 1.9 | 1.2 | 1.6 | 1.6 |
| Severity Scoring | Trace =0.5 | 1+=1.0 | 2+=2.0 | 3+=3.0 |

calcium and urea were normal at the time of the seizures. No confusional states or other neurological abnormalities as reported by others were recorded (2).

b) *Vomiting:* Vomiting occurred in all but 2 courses but was considered comparable in severity to other non-platinum containing regimens used on the unit.

c) A single episode of Radiation Recall occurred in a patient treated with regimen C for Ewings Sarcoma of the scapula. This was of mild/moderate severity with moderate skin erythema and desquamation but with full subsequent recovery.

d) No clinical or biochemical renal or hepatic toxicity was documented. There were no instances of significant hypotension due to VP16 or coagulation disorders as reported by others (1).

### DISCUSSION

Ifosfamide is now established as a first line agent in the treatment of paediatric malignancies. Encouraging results have been obtained in the treatment of poor prognostic sarcomas such as Ewings Sarcoms and advanced Rhabdomyosarcoma (2,3). It is incorporated in current West German and UKCCSG Ewings Sarcoma Trials and the SIOP and Italian Rhabdomyosarcoma Trials. Ifosfamide is also active against other tumours such as advanced or relapsed Wilms Tumour (3,4) and Osteosarcoma (2,3). Responses have been documented in a number of tumours refractory to cyclophosphamide therapy (2,3,4). Our own experience with ifosfamide therapy is similar and will be reported elsewhere. It remains to be seen whether ifosfamide is superior to cyclophosphamide in randomised trials in the treatment of paediatric solid tumours, although preliminary results from a European trial comparing these two agents for adult sarcomass, favour ifosfamide (5).

In this toxicity study, Ifosfamide plus MESNA was well tolerated at a dose of 6 gm/m$^2$, either as a single agent or combined with VP16(213). Although neutrophil counts fell below $0.5 \times 10^9$/L after 56% of courses, there were only 4 (4.2%), instances of febrile neutropaenia and no demonstrable bacteraemias. Only 8% of subsequent chemotherapy courses were delayed due to myelosuppression.

The introduction of regional detoxification with MESNA has ameliorated the hitherto dose-limiting urothelial toxicity. In animal models Habs and Schmahl were able to show that MESNA offered a protective effect against oxazophorine-induced bladder cancer (6). We chose a regimen for MESNA as a 24 hour infusion of either 100% or 150% of the Ifosfamide dosage, preceeded by a relative dose of 16.7% or 25% as a bolus. The MESNA and Ifosfamide infusion was followed by four further boluses totalling 75% or 100% of the Ifosfamide dosage. Post-infusion MESNA boluses were given 3 hourly in contrast to the more usual 4-hourly regimen on discussions with the manufacturers and following the report of Link et al (7). The absence of haematuria recorded in this series is consistent with the findings of Pinkerton et al (4) and Magrath (2), but De Kraker and Voute reported a 16% incidence of frank haematuria using 130% of the total Ifosfamide dosage given as 4-hourly boluses (3). The higher dose of MESNA in our study did not afford protection from microscopic haematuria.

Two generalized convulsions occurred as reported by others (3,4). This appears to be an idiosyncratic phenomenon of uncertain causation. It has been suggested that MESNA could be responsible for promoting seizure activity, but more recent work has implicated toxic Ifosfamide metabolites. We and others (3, 4) were able to administer further Ifosfamide to patients experiencing convulsions without further seizure activity.

The optimum method of ifosfamide administration is not yet established. Further studies are required to compare short and prolonged infusions and to determine whether regimens spread over several days confer an advantage. Larger scale trials are required to define the role of ifosfamide in the treatment of paediatric malignancy and to determine whether it is superior to cyclophophamide. Initial reports however with respect to both efficacy and toxicity seem encouraging and justify randomised trials in which it is used as an agent of first choice in comparison to its more established congener cyclophosphamide.

### REFERENCES

1. *Product Information*—Boehringer Ingleheim.
2. MAGRATH, I. SANDLUND, J. et al (1986). A phase II study of Ifosfamide in the treatment of recurrent sarcomas in young people. *Cancer Chemother Pharmacol* (1986) **18**, (Subbl 2): 525–528.
3. DR KRAKER, J. VOUTE, P. A. (1984). Ifosfamide and vincristine in paediatric tumours. A phase II study. *Eur Paediatr Haematol Oncol* **1**: 47–50.
4. PINKERTON C. R. ROGERS, H. et al (1985). A Phase II study of Ifosfamide in children with recurrent solid tumours. *Cancer Chemother Pharmacol* (1985) **15**, 258–262.
5. VIVIAN, H. BRAMWELL, C. et al (1986). Cyclophosphamide versus Ifosfamide: preliminary report of a randomized phase II trial in adult soft tissue sarcomas. *Cancer Chemother Pharmacol* (1986) **18**, (Suppl 2): 513–516.
6. HABS, M. R. and SCHMÄHL, D. (1983) Prevention of urinary bladder tumours in cyclophosphamide-treated rats by additional medication with the uroprotectors Sodium 2-Mercaptoethane Sulphonate (Mesna) and Disodium 2,2'-Dithio-bis-ethane Sulphonate (Dimesna) *Cancer* **51**, 606–609.
7. LINK, H., NEEF, V., *et al* (1981) Prophylaxis of haemorrhagic cystitis due to cyclophosphamide conditioning for bone marrow transplantation." *Blut* **43**, 329–330.

